# Attitudes Toward Inclusion and Benefits Perceived by Families in Schools with Students with Autism Spectrum Disorders

**DOI:** 10.1007/s10803-022-05491-5

**Published:** 2022-04-23

**Authors:** Cecilia Simón, Gabriel Martínez-Rico, R. A. McWilliam, Margarita Cañadas

**Affiliations:** 1 Department of Developmental and Educational Psychology, Autonomous University of Madrid, Madrid, Spain; 2 Department of Inclusive Education and Socio-Community Development, Catholic University of Valencia, Campus Capacitas, Valencia, Spain; 3 Department of Special Education and Multiple Abilities, The University of Alabama, Tuscaloosa, AL USA; 4 Department of Occupational Sciences, Developmental and Educational Psychology, Catholic University of Valencia, Campus Capacitas, Valencia, Spain

**Keywords:** Learners with ASD, Inclusive education, Attitudes toward inclusion, Benefits of inclusion, Partnership school–family

## Abstract

The aim of this study was to find out about attitudes toward inclusion and benefits perceived by families with children enrolled in schools attended by students with ASD at different educational stages (from kindergarten to high school). 323 families of classmates of students with ASD from different educational stages of 16 mainstream schools participated. The analysis of the attitudes, perceived benefits, relationship with the teacher, and relationship with the school was carried out through questionnaires. The results show positive attitudes toward the inclusive education of students with ASD in all families, but especially among families of children with SEN. All the families identified the benefit of inclusion. Attitudes are related to collaboration with the school and satisfaction with teachers.

## Introduction

Inclusive education is a matter of human rights (European Union Agency for Fundamental Rights, 2020; UN, 2006) rooted in the Universal Declaration of Human Rights (UN, 1948). Moreover, it is a multiplier right, due to its potential to empower people (Human Rights Council, 2019). Inclusion and equity are considered pillars that must support the improvement of schools (UNESCO, 2017), thus constituting an international challenge. This challenge is documented in the 2030 Agenda for Sustainable Development (UNESCO, 2015), although this is not new, according to the Declaration of Salamanca (UNESCO, 1994). The “2020 Global Education Monitoring Report: Inclusion and education: All means all” (UNESCO, 2020a), however, shows the discouraging advances in this international challenge, despite the available evidence on the feasibility of implementing and sustaining improvement towards more inclusive educational systems (UNESCO, 2020b). This report (UNESCO, 2020a) highlights the situation of the most vulnerable groups, including students with disabilities. Among these, it is known that teachers perceive students with autism spectrum disorders (ASD), especially those who require more support, with greater concern regarding their inclusion (Cassimos et al., 2016; de Bruin, 2019; Humphrey & Symes, 2013; González del Rivera, et al., in press). The present work is focused on these students who are vulnerable to processes of educational exclusion.

Nowadays, inclusive education is understood as a moral and judicial imperative (Kefallinou et al., 2020) and a reflection of a fair society (Simón et al., 2019), so it should not require any further justification. As the Right Honourable Helen Clark, Chair of the GEM Report Advisory Board, pointed out, “Debating the benefits of inclusive education can be seen as tantamount to debating the benefits of abolition of slavery or indeed of apartheid” (UNESCO, 2020a, p.9). Moreover, the available evidence indicates that inclusive education benefits not only society, but every student (Kefallinou et al., 2020). It is pertinent to delve into the analysis of these benefits of inclusive education. Do different stakeholders in the educational community, particularly families, perceive benefits the same way? What are the attitudes of families of children with and without disabilities toward the inclusion of students with ASD?

The path towards inclusion requires the support of all stakeholders, and the family is one of these fundamental stakeholders, as has been reported in different countries implementing revision and improvement towards inclusion (Porter & Towell, 2019; UNESCO, 2020b). These concerns guided the present study.

## Who Benefits from Inclusive Education?

The few studies that have been conducted to date on the benefits of inclusion are mainly focused on analyzing the impact on students with and without SEN. Although we agree with the UNESCO (2020a) on the reluctance of using the term “SEN,” we employ it in this article owing to widespread use of the term. We also explored the impact on the teachers and the students’ families. There is little evidence, however, on the perception (toward inclusion) of families of classmates of students with SEN. The literature indicates that learning and developing in schools where all students, including students with disabilities, are welcome has a neutral or positive impact on different stakeholders. According to Gray et al. (2021) the balance of evidence is to neutral or small positive effects as opposed to negative effects and the diversity of factors that play a role in these results must be considered. With respect to teachers, the concern for certain students can involve the implementation of improvements in their teaching practice that ultimately benefit all the students in the classroom (Hehir et al., 2016). Such improvements can be related to greater collaboration among professionals, which positively influences not only classroom practices but also the emotional well-being of the teachers (Drossel et al., 2019). Teachers experience professional growth, increased personal satisfaction, and better self-perception of professional competence (Jordan et al., 2010). Moreover, the available data show that the inclusion of students with disabilities either has no impact on the academic performance of their classmates or, if there is an impact, it is positive (Hehir et al., 2016; Szumski et al., 2017). A positive effect has been reported in the emotional and social development of the classmates of students with SEN (Hehir et al., 2016), facilitating the development of positive attitudes toward inclusion, acceptance, and appreciation of diversity, development of interpersonal skills, and so on (Alnahdi, 2019; Cologon, 2019).

Regarding students with disabilities, important positive impacts have been reported at the academic and social level (Oh-Young & Filler, 2015). Inclusive education can provide a range of academic and social benefits for students with disabilities, such as higher achievement in language and mathematics, improved rates of high school graduation, and more positive relationships with students without disabilities (Hehir et al., 2016). Inclusion has additional benefits (compared to those schooled in special education schools) for their learning and in the maintenance and generalisation of learning between and across settings (Causton-Theoharis et al., 2011). Moreover, academic and communication benefits have been reported, even for students diagnosed with severe and multiple disabilities, as well as a positive behavioural and social development (Hehir et al., 2016). As Kefallinou et al. (2020) pointed out in their meta-analysis, the long-term benefits of inclusive education result in greater chances to continue studying in higher education or finding a remunerated job, compared to students with SEN educated in special education schools.

ASD students with greater support needs face a series of barriers that might prevent them from making the most of their inclusive education (Humphrey & Symes, 2013). Parents who defend the right of their children with ASD to be educated in inclusive environments believe this context provides their children with opportunities to participate with neurotypical peers, which is regarded as an important element in their development (De Boer et al., 2010; Koster et al., 2009).

When inclusive education works, families perceive a positive impact on personal and family well-being (Cologon, 2019). It is worth questioning, however, whether different members of the educational community know about and recognize these beliefs, especially families of classmates. As UNESCO (2020a) highlighted, teachers, teaching materials, and learning environments often ignore the benefits of embracing diversity.

Families can be considered powerful facilitators of inclusion but also potential barriers (Lindsay et al., 2013). Families’ attitudes regarding the inclusion of certain students, such as students with ASD, are linked to perceptions of benefits obtained from inclusion, as has been reported in studies conducted with families of children with SEN (Friedman, 2019).

## Attitudes of Families of Children With and Without Disabilities Toward Inclusive Education

For every learner to feel valued and respected, with a clear feeling of belonging, numerous barriers, rooted in the exclusion processes, still exist (UNESCO, 2020a). Discrimination, stereotyping, and stigmatization are similar for all learners at risk of exclusion (p.10). Moreover, stereotypes have a direct impact on the behaviour of not only teachers but also of classmates and families, influencing other aspects as well, such students’ self-esteem (Braun, 2019).

Families have been fundamental drivers of change toward an increasingly inclusive education and an essential stronghold in the struggle for the rights of their children, particularly regarding their education (Echeita & Simón, 2021). Similarly, “Parents may hold discriminatory beliefs about gender, disability, ethnicity, race or religion. Some 15% in Germany and 59% in Hong Kong, China, feared that children with disabilities disturbed others’ learning” (UNESCO, 2020a, p. 21).

Attitudes toward inclusion from different members of the educational community constitute one of the important facilitators or barriers in the process of improvement of schools aiming to become more inclusive (De Boer et al., 2012; Rodríguez et al., 2012). Although the available evidence shows the importance of families of classmates without SEN in this process, particularly their attitudes (De Boer et al., 2012) few studies have addressed their attitudes toward inclusion (Albuquerque et al., 2019; De Boer et al., 2010; Paseka and Schwab, 2020). For example, when families do not have positive attitudes toward inclusion, (i.e., when they do not support inclusion), they may negatively influence their children’s attitudes and behaviour (De Boer et al., 2012); thus, these attitudes become indirect factors affecting social relationships among students (Sosu & Rydzewska, 2017; Zanobini, et al., 2018). They can also become stress factors for families of children with SEN (Cologon, 2019).

According to Sosu & Rydzewska (2017), parental attitudes toward inclusion are rooted in beliefs about the impact on their children’s education. The present study, in line with previous studies that analysed attitudes regarding students with SEN (De Boer & Munde, 2015; Sira et al., 2018), is based on Triandis’s (1971, p. 266) proposition that attitudes are a “learned predisposition reflecting how favorable or unfavorable people are toward other people, objects or events, and they have three components: cognitive, affective, and behavioural”. The cognitive component refers to the knowledge about the capacities and performance of children with disabilities. The affective component is related to the understanding of disability, which can lead to accepting or refusing to work with a person with a disability. The behavioural component is related to the tendency of an individual to respond in a certain way to a situation in which people with disabilities are involved. Thus, (a) the cognitive component would be related to families’ beliefs (e.g., about the rights of children with disabilities to be educated in general schools); (b) the affective component would show their feelings and concerns (e.g., about the implications of the inclusion of students with disabilities in the classroom of their children); and (c) the behavioural component would reflect the family’s actions or ways of behaving in certain situations (e.g., inviting a child with a disability to their child’s birthday party).

Although families of children with disabilities value inclusion and its potential for the academic and social development of their children, they are concerned about a set of associated factors that can limit children’s presence, learning, and participation (Ainscow et al., 2006; Booth & Ainscow, 2011). Such factors are (a) the training of teachers to understand and respond to the educational needs of all students, ensuring an individualized attention (Abu-Hamour & Muhaidat, 2014; Hotulainen & Takala, 2014; Rogers, 2007; Yssel et al., 2007); (b) the lack of resources and supports required by teachers to reach all students (De Boer et al., 2010; Hotulainen & Takala, 2014; Rogers, 2007; Villegas et al., 2014); (c) the delegation of the responsibility for educating their children to “specialized” teachers, which poses the need to revise the understanding and modes of support provided, which in turn requires adequate coordination and collaboration among the different professionals and services (Gasteiger-Klicpera et al., 2013; Villegas et al., 2014); (d) the type of psycho-pedagogical evaluations founded on a medical model, which are far from the concept of inclusive evaluation (Simón et al., 2021); (e) the lack of effective leadership in schools (Kluth et al., 2007), as well as a school culture not based on inclusive values (Villegas et al., 2014); and (f) the development of academic competencies and attention to the social and emotional well-being of their children to prevent bullying, which is a special concern in secondary education (Abu-Hamour & Muhaidat, 2014; de Boer et al., 2010; Hotulainen & Takala, 2014; Rogers, 2007; Yssel et al., 2007).

Also, these concerns can vary depending on different factors such as the level of support that their children require. In this sense, some studies have reported significant differences in attitudes toward inclusion, depending on the severity of the ASD: Families of children with *signs* of ASD obtained a higher score on attitude scales than families of children *with* ASD and great support needs (Su et al., 2016, 2020). In the study of Su et al. (2020), most of the families of ASD children (87.8%) agreed or strongly agreed with the idea that their children must go to general classrooms, whereas only 55.8% of the families of typically developing children agreed or strongly agreed with the idea that children with ASD can study in general classrooms.

Families of children without disabilities recognize the benefit of educational inclusion for the development of certain socioemotional competencies in their children, in addition to values related to valuing diversity (Most & Ingber, 2016; Sira et al., 2018; Vlachou et al., 2016). In some studies, however, families state these benefits are greater for students with disabilities than for those without disabilities (Sosua & Rydzewska, 2017). They are concerned about the attention teachers’ pay to their children (Albuquerque et al., 2019), the training of teachers to manage the classroom (Albuquerque et al., 2019; De Boer & Munde, 2015), the possibility that teachers dedicate too much time and attention to the children with disabilities, and the probable deterioration of the quality of teaching (Albuquerque et al., 2019).

Therefore, families in general, and those with children with disabilities in particular, need to trust that ordinary schools will respond to their needs and ensure the well-being of their children. Focusing on families of children with ASD, however, expectations by schools increase as children age, and thus families of older children look for schools that better meet their needs (UNESCO, 2020a). Some studies conducted in different countries show that attitudes of families with typically developing children toward the educational inclusion of children with disabilities are neutral (Albuquerque et al., 2019; De Boer et al., 2012) or positive (Hu et al., 2018; Most & Ingber, 2016; Vlachou et al., 2016; Sosua & Rydzewska, 2017), although these vary depending on certain factors related to students and families.

Some of the factors regarding the attitudes of families with children without SEN toward inclusion are related to families themselves while others are linked to children: a: (a) The *gender* of the parents*,* no conclusive results have been found. Some studies have not detected significant differences between fathers and mothers (Freitas et al., 2015), whereas others have reported more positive attitudes among mothers (de Boer & Munde, 2015; de Boer et al., 2012): (b) The *education level* of the parents. Whereas some studies have not found this factor to be related to attitudes (de Boer & Munde, 2015; de Boer et al., 2012), others have found more positive opinions among mothers with higher education levels (Sosua & Rydzewska, 2017; Su et al., 2020); (c) *Age*. Some studies show that younger parents have more positive attitudes than do older parents (de Boer & Munde, 2015), whereas other studies report opposite results (Sosua & Rydzewska, 2017); (d) *Socioeconomic situation.* Some researchers have associated lower family income with positive generalised beliefs (Sosua & Rydzewska, 2017), whereas others have reported that families in a medium–high socioeconomic situation have more positive attitudes (Most & Ingber, 2016); (e) *Experience with inclusive education*. In some cases, experience with inclusion has been related to more positive attitudes (Kalyva & Agaliotis, 2009). Most & Ingber (2016) found this correlation in families with a high socioeconomic level, but not in those with a low socioeconomic level: (f) *Contact with people with disabilities* as a facilitator of more positive attitudes (Alburquerque et al., 2019; de Boer & Munde, 2015); (g) *Personality traits* (Albuquerque et al., 2019). Albuquerque et al., (2019, p.378) found significant associations between personality dimensions and parental attitudes (“agreeableness and conscientiousness were significantly associated with more positive attitudes towards inclusion, while neuroticism showed negative associations”); (h) *Satisfaction with the school.* A high level of satisfaction with the school in which the child is educated is associated with positive beliefs (Sosua & Rydzewska, 2017); (i) *Having a child with SEN.* In some cases, a positive relationship in attitudes toward inclusion has been found (De Boer & Munde, 2015; De Boer et al., 2012), whereas, in other cases, such associations did not exist (Rafferty & Griffin, 2005); (j) *Type of disability.* Attitudes differ by type of disability, being more favourable toward inclusion in the case of children with sensory disabilities (Albuquerque et al., 2019; Hilbert, 2014) or motor disabilities (De Boer & Munde, 2015), and less favourable in the case of children with behavioral disorders, intellectual and developmental disabilities (Albuquerque et al., 2019; de Boer & Munde, 2015; Freitas et al., 2015; Hilbert, 2014), or children with profound intellectual and multiple disabilities (de Boer & Munde, 2015).

Families of both typically developing children and children with disabilities regard the relationship with teachers as fundamental for the success of inclusion (Guralnick, 2011). Parental support of inclusion may depend upon their attitudes, reactions, and experiences related to classroom practices (Sira et al., 2018). Families need to feel supported by the teacher of their children and by other families and they also require information (the benefits of inclusion, how to communicate effectively with and respond to the children, etc.). Moreover, the communication between teachers and families facilitates a trust relationship with the teacher and a positive relationship among the families (Sira et al., 2018). Therefore, the variables “collaboration with the teacher” and “collaboration with the school” are factors considered in this study.

Based on all of the above-mentioned background, the objectives of the present study were the following: (a) to know the attitudes of families (both with children with SEN and those of their classmates) whose children go to school with children with ASD; (b) to know what benefits they perceive from inclusive education; (c) analyze the relevance of family factors, such as having a child with SEN and the educational stage of their children, in attitudes toward the inclusion of students with ASD; and (d) to study the relationship between perceived attitudes/benefits and teacher factors (relationship with the family) and school factors (school–family alliance).

## Method

### Participants

A total of 323 families with children at different educational levels (from early childhood education to baccalaureate) and families of classmates of students with ASD from 16 mainstream schools participated in this study. The criteria for inclusion at schools were the following: (a) to have students with ASD in the school (all the early childhood, primary, and secondary schools are targeted for the schooling of students with ASD) and (b) that the students with ASD spend more than 50% of the school day in the regular classroom. In addition, the 16 schools that finally participated in the study have made a commitment to inclusion and continuous assessment an important topic in their educational project. Furthermore, the teachers' perceptions and concerns for the learning and participation of students with ASD has been confirmed in a parallel study carried out with these schools (EDITEA, 2021).

127 families had children in early childhood education, 90 in primary education, 61 in secondary education, and 45 in baccalaureate. The children of the families who responded to the questionnaire have direct contact with pupils with ASD (classes, playground, extracurricular activities).

Regarding disabilities, 90 families had children with special needs, and 233 families had children without special needs. Table 1 shows the characteristics of the participating families.

**Table 1:** Characteristics of the families

Characteristic	Whole sample *n* (%)	Without SEN *n* (%)	With SEN *n* (%)
*Have children with SEN*			
No	233 (72.1)		
Yes	90 (27.9)		
*Educational stage of the child*			
Early childhood	127 (39.3)	62 (26.6)	65 (72.2)
Primary	90 (27.9)	76 (32.6)	14 (15.6)
Secondary	61 (18.9)	54 (23.2)	7 (7.8)
Baccalaureate	45 (13.9)	41 (17.6)	4 (4.4)
*Gender of the child*			
Male	40 (12.4)	0 (0)	40 (44.4)
Female	13 (4.0)	0 (0)	13 (14.4)
Missing values	270 (83.6)	233 (100)	37 (41.4)
*Contact with other SEN people*			
No	197 (61.0)	134 (57.5)	63 (70.0)
Yes	53 (16.4)	37 (15.9)	16 (17.8)
Missing values	73 (22.6)	62 (26.6)	11 (12.2)
*Parent*			
Mother	61 (18.9)	50 (21.5)	11 (12.2)
Father	15 (4.6)	10 (4.3)	5 (5.6)
Both	56 (17.3)	0 (0)	56 (62.2)
Missing values	191 (59.1)	173 (74.2)	18 (20.0)
*Age of the mother*			
20–29 years	1 (0.3)	1 (0.4)	0 (0)
30–39 years	10 (3.1)	5 (2.1)	5 (5.6)
40–49 years	93 (28.8)	46 (19.7)	47 (52.2)
50–59 years	110 (34.1)	88 (37.8)	22 (24.4)
> 60 years	49 (15.2)	47 (20.2)	2 (2.2)
Missing values	60 (18.6)	46 (19.7)	14 (15.6)
*Education level of the mother*			
No studies	1 (0.3)	1 (0.4)	0 (0)
Primary	10 (3.1)	7 (3.0)	3 (3.3)
Secondary	25 (7.7)	16 (6.9)	9 (10.0)
Vocational training	70 (21.7)	47 (20.2)	23 (25.6)
Baccalaureate	159 (49.2)	117 (50.2)	42 (46.7)
Missing values	58 (18.0)	45 (19.3)	13 (14.4)
*Age of the father*			
20–29 years	4 (1.2)	1 (0.4)	3 (3.3)
30–39 years	18 (5.6)	8 (3.4)	10 (11.1)
40–49 years	28 (8.7)	19 (8.2)	9 (10.0)
50–59 years	20 (6.2)	16 (6.9)	4 (4.4)
> 60 years	1 (0.3)	1 (0.4)	0 (0)
Missing values	252 (78.0)	188 (80.7)	64 (71.1)
*Education level of the father*			
No studies	1 (0.3)	1 (0.4)	0 (0)
Primary	2 (0.6)	2 (0.9)	0 (0)
Secondary	6 (1.9)	2 (0.9)	4 (4.4)
Vocational training	10 (3.1)	7 (3.0)	(3.3)
Baccalaureate	52 (16.1)	33 (14.2)	19 (21.1)
Missing values	252 (78.0)	188 (80.7)	64 (71.1)

SEN children without (No) or with (Yes) special education needs

### Instruments

– *Questionnaire on the attitudes of parents toward inclusive education*, translated and adapted from De Boer et al. (2012). This questionnaire is a self-report questionnaire consisting of a vignette that describes a hypothetical child with ASD and 23 attitude statements. These statements evaluate three components of attitudes described in the introduction: cognition (13 items), affective (6 items), and behaviour (4 items). After reading the vignette, the parents were asked to indicate their degree of agreement with the attitude statements based on a 4-point Likert scale (1 = totally disagree, 4 = totally agree), with a maximum score of 92 points. A higher score reflects a more positive attitude.

Once the authors gave their permission, a bilingual person was given the questionnaire to translate it, attending to cultural aspects. A reverse translation was conducted, following the guidelines of Balluerka et al. (2007). Item 2 was removed from the attitudes questionnaire for being negatively correlated with the rest of the items. Last, a reliability analysis was conducted, both for the original questionnaire (α = 0.89), indicating very good reliability (George & Mallery, 2003), and for the subscales (cognition: α = 0.83; affective: α = 0.79; behaviour: α = 0.51). The behaviour subscale has a low reliability, the implications of this will be pointed out in the discussion and in the limitations.

– *Questionnaire on the perception of the benefits of inclusive education*. The research team designed this questionnaire for this study. It consists of 16 items evaluating two areas: advantages and disadvantages for classmates of learners with ASD (6 ítems) and for teachers and the school (10 ítems). This questionnaire is completed by indicating the level of agreement with the statements presented in the items based on a Likert scale, with 5 options (1 = totally disagree, 5 = totally agree). The maximum score that can be obtained is 80 points. This questionnaire was validated by a group of experts (researchers related to inclusive education and families), who were asked to assess the appropriateness, relevance, and clarity of each item. A rating scale of 1–3 points was used (1 = not at all appropriate/not relevant/not clear; 2 = not very appropriate/not very relevant/not very clear; 3 = very appropriate/very relevant/very clear). As a result of this process, two items were removed from the questionnaire. This decision was based on the exclusion criterion of those items that were rated with 2 or fewer points in their relevance or appropriateness. None of the items were considered “not clear” or “not very clear”. The reliability analysis produced good results for both dimensions of this questionnaire: advantages for classmates (α = 0.853) and advantages for teachers and school (α = 0.791). The Cronbach’s α for the whole questionnaire was 0.89.

– *Questionnaire on the power of alliances with the families.* This is a scale adapted from The Power of Partnerships Family Survey (National PTA Standards for Family-School Partnerships, 2009). This scale evaluates the relationship/participation between families and school and it consists of 21 items, scored with a Likert scale based on the extent of agreement with the statements presented (1 = totally agree, 5 = totally disagree). These 21 items assess the following dimensions: (a) welcoming families to the school; (b) maintaining effective communication; (c) supporting students’ success; (d) speaking to, in favour of, and up for every child; (e) sharing power; and (f) collaborating with the community. Cronbach’s α of the whole questionnaire was 0.95.

– *Family-Professional Partnership Scale of the Beach Centre on Disability* (Summers et al., 2005), adapted by Balcells-Balcells et al. (2016). This scale assesses whether the collaborativeness of the relationship established by the professional with families. We considered the scale suitable for evaluating the specific actions of the classroom teacher, with the addition of an item related to a relevant aspect of the role of the teacher: “The “teacher” has information about my child from the rest of the teachers”. This questionnaire consists of 19 items, with good reliability values (α = 0.96). The scale evaluates satisfaction, focused on actions with the child (α = 0.848) and the family itself (α = 0.926).

– *Protocol of sociodemographic data.* This protocol consists of questions for describing the participants, regarding both the caregivers (age, gender, education level, etc.) and their children (course of the child, child with or without SEN, etc.).

### Procedure

The families who volunteered to participate in the study answered the questionnaire in paper or online format. The questionnaires were anonymous, guaranteeing the confidentiality of the information. This study was approved by the Research Ethics Committee of the university to which the first author belongs.

### Data Analysis

First, descriptive analyses were performed for the items and total scores of the different questionnaires. Second, the unidimensionality of the questionnaires was studied through the ratio between the first and second self-value, along with the mean communality and the square correlation of the factor scores from a unidimensional factor analysis. The Habeman strategy was also applied (Reise et al., 2013) to verify whether the total mean of the questionnaires of attitudes and benefits was a good indicator of the possible theoretical subscales. Once the unidimensionality of the questionnaires was verified, the reliability was checked through Cronbach’s alpha.

All the questionnaires obtained an alpha above 0.89, a mean communality of the items higher than 0.54, a ratio between the first and second self-value over 3.89, and a reliability of the factor scores greater than 0.92.

A Haberman analysis (Reise et al., 2013) was carried out to verify whether a unidimensional factor analysis would allow an accurate prediction for scores in each subscale as a factor analysis with three factors (for attitudes) and with two factors (benefits). All of these results justify the use of the questionnaire global score in subsequent analyses.

**Fig. 1:**
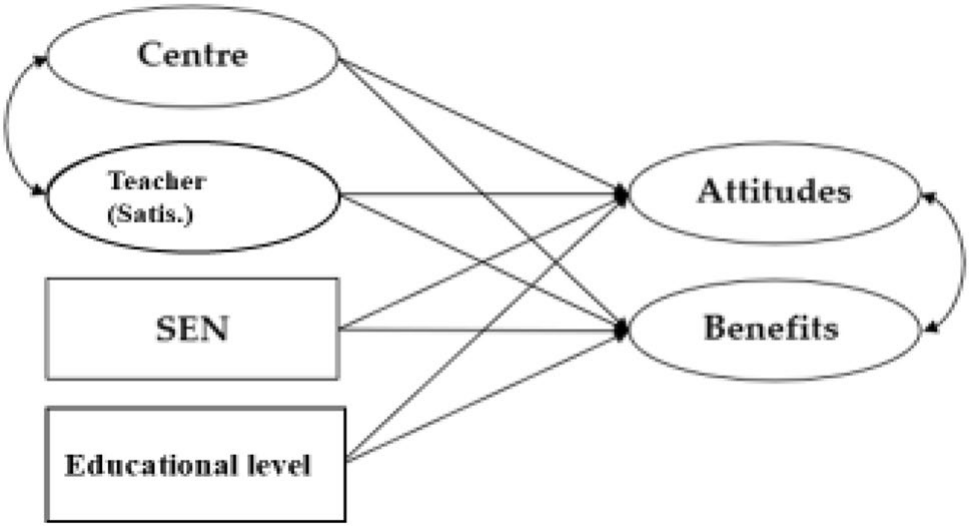
Model of structural equations. SEN children without (No) or with (Yes) special education needs

To study the relationships between the variables *SEN* (having a child with SEN) and *Educational stage* through the questionnaires of attitudes and benefits, two ANOVAs of two fully randomised factors were conducted. The assumption of homoscedasticity was verified in both cases. Additionally, a model of structural equations was performed, which included, in addition to *SEN* and *Educational stage*, the scores obtained in the *Centre* and *Satisfaction with the classroom teacher* questionnaires (Fig. 1).

Because the items of the questionnaires were categorical, the robust estimator of diagonally weighted mean squares (WLSMV) was used. The fit of the model was verified using CFI, TLI and RMSEA. The analyses were conducted in R statistical software (R Core Team, 2020), using the *psych* package (Revelle, 2019). The model of structural equations was estimated with Mplus (Muthén & Muthén, 2020), using the *MplusAutomation* package (Hallquist & Wiley, 2021) of R.

## Results

The main aim of this study was analyse attitudes about inclusion and perceived benefits to families of children with and without SEN enrolled in ordinary schools with ASD students. Table 2 shows the means of the questionnaires of attitudes and benefits, as well as those of the subscales.

**Fig. 2:**
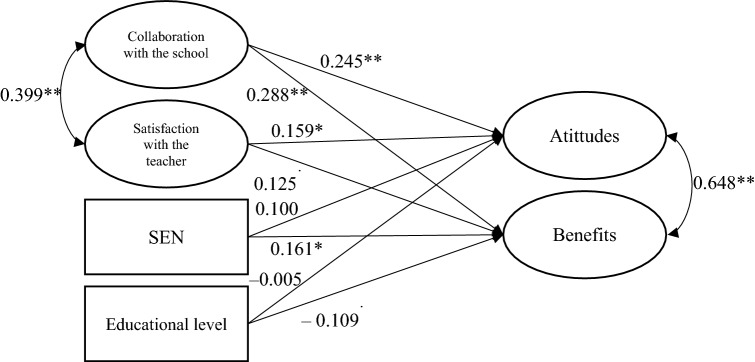
Relationship between SEN, educational level, family-teacher satisfaction and family-school collaboration. SEN children without (No) or with (Yes) special education needs

- Attitudes of families towards the inclusion of students with ASD
  One objective of the study focused on analysing the attitudes of families towards the inclusion of pupils with ASD, and whether these attitudes vary according to whether or not they have a child with SEN. Positive attitudes towards inclusion were found in all families who participated in the study (see Table 2). However, the results indicate that, in general, families of children with SEN had more positive attitudes toward inclusive education (*p* = 0.0050; ${\eta }_{p}^{2}$ = 0.016) than did families of children without SEN; that is, they reflected a more receptive and favourable attitude toward the inclusion of ASD students with great support needs in schools. Scores were higher in the cognitive, affective, and behavioral components for families of children with SEN, showing significant differences only in the cognitive component (*p* = 0.032; ${\eta }_{p}^{2}$ = 0.018). This seems to indicate that families of children with SEN believed students with ASD with great needs should be included in ordinary schools and, thus, have the same rights as typically developing children. This result is further discussed with the structural equation analysis.
- Perceived benefits for families
  The second objective of this research analyses the perceived benefits for families with and without children with SEN. Both families identify numerous advantages for all learners. Those that have been highlighted are: “In inclusive schools, children are more likely to respect and value human differences” (X ® = 4.44; *SD* = 0.887); “Working with learners with specific educational support needs invites teachers to train and work collaboratively with others” (X ® = 4.33; *SD* = 0.744); “Being educated in inclusive schools better prepares all children (with and without specific educational support needs) for the real world” (X ® = 4.29; *SD* = 1.007); “Having students with specific educational support needs helps teachers to reflect on their practices and try to improve it so that all students learn” (X ® = 4.24; *SD* = 0.810); “Relationships with families in inclusive settings are more collaborative” (X ® = 4.24; *SD* = 0.934); “In inclusive settings, families are seen as a problem rather than as a support for the classroom and setting” (X ® = 4.21; *SD* = 0.957); “In inclusive settings, students with specific educational support needs have more opportunities to promote their personal and social development” (X ® = 4.21; *SD* = 0.975); “In classrooms where there are students with specific educational support needs, teachers must introduce changes in their teaching practice that benefit all students” (X ® = 4.16; *SD* = 0.924).
  Results show significant differences both in the total scale (*p* = 0.001; ${\eta }_{p}^{2}$ = 0.042) and in the two subscales related to perceived benefits (see Table 2): (a) the benefits of inclusion for the classmates (i.e., for all students, *p* = 0.013; ${\eta }_{p}^{2}$ = 0.020) and (b) the benefits for the school and the teachers (*p* < 0.001; ${\eta }_{p}^{2}$ = 0.049). In general, families of children with SEN perceived greater benefits of schooling in regular schools including students with ASD with great support needs ($\overline{X }$ = 67.79; *SD* = 8.7), compared to families of children without SEN ($\overline{X }$ = 63.35; *SD* = 9.4; *p* < 0.05). Thus, they consider inclusive education to be a value for everyone. They believe that the advantages of inclusion are greater for all students as a whole ($\overline{X }$ = 25.82; *SD* = 4.3 for the families of children with SEN vs. $\overline{X }$ = 24.46; *SD* = 4.7 for the families of children without SEN), regardless of whether the students have specific educational support needs or not. Furthermore, it was again the families of children with SEN who showed a more positive perception of inclusive education, believing the benefits of inclusion were also positive for the school and the teachers ($\overline{X }$ = 41.88; *SD* = 5.2), whereas the families of children without SEN had a lower mean score ($\overline{X }$ = 38.89; *SD* = 5.5; *p* < 0.05).
- Attitudes and benefits according to educational stage
  Having analysed the attitudes of families towards the inclusion of pupils with ASD in the classroom as well as the benefits perceived by families, this research has focused on analysing whether there are differences between the different educational stages. The analysis of the attitudes and benefits perceived by families according to educational stages also shows a significant result. The ANOVA results show significant differences in the emotional component of attitudes towards inclusion between the different educational stages (see Table 3). The post-hoc Tukey's pairwise analyses, however, did not reach the necessary significance level for any of the educational stages.
- Family collaboration with the school and satisfaction with the teacher
  This study also explored whether attitudes toward inclusion and perceived benefits were affected by the extent of collaboration between the school and families of children with and without SEN, as well as by the extent of satisfaction of families with the classroom teacher. Data show that families of children with SEN establish a relationship of greater collaboration with the school than do families of children without SEN ($\overline{X }$ = 90.34; *SD* = 13.17) (Table 4), with *t* (267) = 3.374 (*p* < 0.001; *d* = 0.41; 95% CI = 0.17, 0.66), and assuming equal variances according to the Levene test (*F* = 0.434; *p* = 0.511). Similarly, results show that no significant differences regarding the degree of satisfaction with the classroom teacher exist between families of children with SEN and those of children without SEN (Table 4), with *t* (169.9) = 1.285 (*p* = 0.200; *d* = 0.20; 95% CI = − 0.10, 0.50).
- Family-school collaboration and satisfaction with the teacher depending on the stage of education
  These variables were studied as a function of the educational level. Results indicate the different levels differed (Table 5), after comparing the degree of collaboration between the families and the school, *F* (3) = 6.317 (*p* < 0.001; ${\eta }_{p}^{2}$ = 0.067). In general, earlier educational stages showed higher collaboration with the school. The following significant pairwise differences (Tukey) were found: early childhood with secondary (*p* = 0.043; *d* = 0.46; 95% CI = 0.12, 0.79), early childhood with baccalaureate (*p* < 0.001; *d* = 0.76; 95% CI = 0.37, 1.12) and primary with baccalaureate (*p* = 0.022; *d* = 0.53; 95% CI = 0.15, 0.91), showing a moderate effect size. An additional two-way ANOVA has been conducted at both educational level and of families of children with/without SEN regarding the collaboration with the school to further explore whether the previous results were caused by the different distributions of families with/without SEN at different educational levels. The results revealed that this was not the case, as educational level still showed a significant effect [*F*(3) = 6.458; *p* < 0.001; ${\eta }_{p}^{2}$ = 0.069], as well as having children with SEN or not [*F*(1) = 4.157; *p* = 0.042; ${\eta }_{p}^{2}$ = 0.016]. The interaction between both variables did not produce a significant effect.
  Regarding the satisfaction of the families with the classroom teacher as a function of the educational stage, significant differences were also obtained (see Table 5). Families of children in early childhood and primary education showed more satisfaction with the teacher compared to the families of children in secondary education and baccalaureate, with *F*(3) = 10.80 (*p* < 0.001; ${\eta }_{p}^{2}$ = 0.138). The following significant pairwise differences (Tukey) were obtained: early childhood with secondary (*p* < 0.001; *d* = 0.85; 95% CI = 0.40, 1.11), early childhood with baccalaureate (*p* < 0.001; *d* = 1.22; 95% CI = 0.66, 1.48), primary with secondary (*p* < 0.001; *d* = 0.85; 95% CI = 0.42, 1.17) and primary with baccalaureate (*p* < 0.001; *d* = 1.23; 95% CI = 0.68, 1.54), showing a large effect size.
- Relationship between study variables: Structural equation model
  Up to this point, we have analysed the attitudes of and benefits perceived by families of children schooled in schools with students with ASD. In the analysis, we compared families of children with and without SEN, describing the relationship between attitudes toward and benefits of inclusion and two variables potentially relevant in the interpretation of the results: (a) the extent of collaboration between the family and the school, and (b) the extent of satisfaction of the family with the classroom teacher. Likewise, sociodemographic variables were explored: gender of the parent/guardian, education level, and economic situation. None of the analyses showed significant differences in the attitudes or perceived benefits according to these variables.
  The previous analyses were completed with a structural equation model (SEM), to analyse the relationships between the independent variables (Fig. 2). The fit of this model was acceptable: CFI = 0.94; TLI = 0.93; RMSEA = 0.04. The model showed a significant effect of the variable “families of children with and without SEN” on the benefits perceived by the families (0.161; *p* = 0.011), indicating that families of children with SEN had a more positive perception of the benefits of inclusion than did families of children without SEN.
  Relevant results were also obtained in the analysis of the relationship between collaboration with the school and satisfaction of the family with the classroom teacher. The greater the collaboration with the school the better the attitudes toward inclusion and its perceived benefits. The greater the satisfaction with the teacher, the better the attitudes toward inclusion. As can be observed in the model, attitudes and perceived benefits were related to each other (*r* = 0.648; *p* < 0.001). Moreover, the extent of families’ satisfaction with the teacher and the collaboration with the school were also interrelated (*r* = 0.399; *p* < 0.001), that is, the greater the satisfaction with the teacher the greater the collaboration with the school and vice versa.

**Table 2:** Attitudes and perceived benefits of families of children with and without SEN

Dependent variable	SEN		Total sum	Total mean*
	No	Yes		
Attitudes (Total)	76.29 (9.4)*	78.68 (10.1)*	76.98 (9.7)	3.35
Attitudes (Cognitive)	41.28 (5.9)*	42.56 (6.0)*	41.66 (5.9)	3.20
Attitudes (Affective)	21.32 (2.9)	21.86 (3.1)	21.47 (3.0)	3.58
Attitudes (Behavioural)	13.60 (1.8)	13.95 (2.0)	13.70 (1.8)	3.43
Benefits (Total)	63.35 (9.4)*	67.79 (8.7)*	64.59 (9.4)	4.04
Benefits (Classmates)	24.46 (4.7)*	25.82 (4.3)*	24.83 (4.6)	4.14
Benefits (School)	38.89 (5.5)*	41.88 (5.2)*	39.73 (5.5)	3.97

SEN children without (No) or with (Yes) special education needs

*Significant differences

**Table 3:** Attitudes and perceive benefits of the families according to the educational level

Dependent variable	Educational stage			
	Early Childhood	Primary	Secondary	Baccalaureate
Attitudes (Total)	77.09 (11.6)	78.90 (7.8)	75.73 (8.2)	75.17 (9.1)
Attitudes (Cognitive)	41.88 (6.7)	42.63 (5.3)	41.00 (4.9)	40.33 (6.2)
Attitudes (Affective)*	21.29 (3.5)	22.00 (2.3)	21.11 (2.8)	21.43 (2.8)
Attitudes (Behavioural)	13.65 (2.1)	14.05 (1.5)	13.45 (1.8)	13.49 (1.6)
Benefits (Total)	65.86 (9.1)	66.65 (8.6)	62.07 (7.2)	60.89 (12.2)
Benefits (Classmates)	25.31 (4.6)	25.47 (4.4)	23.86 (3.9)	23.62 (5.6)
Benefits (School)	40.52 (5.4)	40.93 (4.9)	38.32 (4.4)	37.27 (7.1)

Attitudes, response scale of 1–4; Benefits, response scale of 1–5

*Significant differences for the effect of the educational stage. There were no differences between pairs of educational stages according to Tukey’s pairwise analyses

**Table 4:** Collaboration of the families with the school and satisfaction with the classroom teacher

	SEN	*N*	Mean	SD
Centre	Yes	74	90.34	13.17
	No	195	84.00	13.97
Teacher	Yes	75	90.69	6.78
	No	203	89.41	8.76

SEN children with (Yes) or without (No) special education needs

**Table 5:** Collaboration of the families with the school and satisfaction with the classroom teacher based on the educational stage

	Stage	*N*	Mean	SD
Centre	Early childhood (0–6 years)	101	89.23	12.59
	Primary (6–12 years)	70	86.79	14.83
	Secondary (12–16 years)	54	83.17	13.74
	Baccalaureate (16–18 years)	44	79.25	13.75
Teacher	Early childhood	106	92.00	5.25
	Primary	77	91.92	4.77
	Secondary	53	85.64	11.46
	Baccalureate	42	85.33	10.99

## Discussion

The aim of this study was to analyse the attitudes about the inclusion of students with ASD and the perceived benefits in families of children enrolled in regular schools with ASD students. We also explored whether these factors are influenced by two important aspects in inclusive education: the extent of collaboration between the school and families of children with and without SEN and the extent of satisfaction with the classroom teacher.

Positive attitudes were generally found in attitudes toward the inclusive education of students with ASD, especially in the families of children with SEN compared to the families of children without SEN (Most, & Ingber, 2016; Sosua & Rydzewska, 2017; Vlachou et al., 2016). This result is consistent with those found by De Boer et al. (2012) and De Boer & Munde (2015). About the different components of attitudes toward inclusion (i.e., cognitive, affective, and behavioural components), families of children with SEN are more confident about the possibilities and capacities of students with ASD and are more supportive of their right to an inclusive education (cognitive component). Both groups of families had similar beliefs about the affective component. Let us remember that these are families who know that their children share educational spaces with students with SEN, including ASD students, which shows that these attitudes are also founded on the knowledge of the reality they live. Moreover, this also shows the need to increase the efforts to provide information to families about the right to an inclusive education and the value of diversity in the classroom.

All the families identified numerous benefits of inclusion, both for students with SEN and for students without SEN. Families of children with SEN, however, perceived even more benefits than those of children without SEN. Families highlighted benefits related to the development of values such as respect for human differences and preparation for the real world, which is in line with previous studies (Alnahdi, 2019; Cologon, 2019; Most & Ingber, 2016; Sira et al., 2018; Vlachou et al., 2016). Families recognised positive effects on the development of social and personal skills (Kefallinou, et al., 2020; Oh-Young & Filler, 2015).

Families of children with SEN consider benefits of inclusion to be more positive for the school and for the teachers than do families of children without SEN, which is in line with the findings of previous studies conducted with other groups (Hehir et al., 2016). They also believe in the benefits of inclusion for all students (with and without SEN).

In this sense, they consider inclusive schools to be prepared to respond to the specific needs of all students and that the teachers are reflective and innovative in their educational practices. Furthermore, they pointed out that the work between teachers is collaborative and participatory and that the relationship between the school and the family is also collaborative.

According to Most & Ingber (2016), teachers and principals must be aware of the different attitudes that parents might have toward inclusion as well as their role. They must provide information and support to families when children without disabilities are educated in an inclusive classroom. Families must know the benefits their children can acquire with the inclusion programmes. Working closely with parent advocates in an inclusive setting is necessary to guarantee the success of this approach (Garrick-Duhaney & Salend, 2000; Leatherman & Niemeyer, 2005).

In this study, we drew conclusions about the two variables related to families and the school. Both variables are key in inclusive schools, as different authors have pointed out (Guralnick, 2011). The collaboration of families with the school and with their children’s teacher, when attitudes and perceived benefits are positive, confirm results obtained in previous studies (Sira et al., 2018). In our study, as expected, these variables are related; thus, the higher the satisfaction with the teacher, the greater the collaboration with the school.

It is worth highlighting that the families of children with SEN report high collaboration with the school and with the classroom teacher. These collaborative attitudes were, in part, predictable, since they are identified with principles of inclusive education and are consistent with the type of school in which the investigation was carried out. Otherwise, we would question the consistency between the values declared and the actions conducted. Both the families of children with SEN and those of children without SEN show similar levels of satisfaction with the teacher. The families of children with SEN, however, establish a more collaborative relationship with the school than do the families of children without SEN. In these families, the greater and more participatory the collaboration with the school, the greater the perceived benefits of inclusive education, which is in line with previous studies (Maich et al., 2019; Perry et al., 2020). Satisfaction with the relationship with the teacher, however, is similar between families of children with SEN and those of children without SEN. The extent of collaboration with the school decreases with the advancing educational levels, being greater in early childhood education compared to secondary education and baccalaureate, and in primary education compared to secondary education and baccalaureate. Similarly, families of children in early childhood and primary education show greater satisfaction with the classroom teacher than do families of children in secondary education and baccalaureate. According to Leyser & Kirk (2004), this may be the result of different factors, both pupil-related (e.g. the increase in pupil self-reliance) and school organisation (e.g. the increase in the number of teachers involved in the development of the school curriculum, which increases from secondary school onwards, diluting the reference figure of families).

These results, in agreement with findings of Francis et al. (2016), strengthen the importance of working with families to provide them adequate information about inclusion, why it is a right, and available data about its impact on students, teachers, and the school (Schuelka et al., 2019). As Sosu & Rydzewska (2017) stated, from the analysis of previous studies, parents express their concern about inclusive education when they perceive that schools do not have the necessary resources, infrastructure, knowledge and capacities. Therefore, the trust of parents in the school must be increased. Information, spaces for reflection and sensitisation, and the involvement of the families in the processes of evaluation and improvement are important. Indeed, this is one of the areas of review in identifying barriers and facilitators for schools that want to become increasingly inclusive (Booth & Ainscow, 2011).

Our study shows that, when the collaboration with the school is higher, both the attitudes toward inclusion and the benefits perceived from it are also greater. Similarly, the greater the satisfaction with the teacher, the more positive the attitudes toward inclusion. These results confirm the relevance of creating trust relationships with families and nurturing their participation in the activities of the school and in decision making related to the school and to their children. This result is reflected in the perception of the benefits of inclusion and in positive attitudes toward the inclusion of ASD students, in particular.

This study has *limitations* that must be pointed out. Firstly, although one of the advantages of this study is the fact that all the participating schools are those schools concerned with inclusion, the results do not allow us to determine whether the findings from these schools are different from what we might expect in schools with no experience in inclusion. Secondly, the low reliability of the behaviour subscale of the questionnaire on the attitudes of parents towards inclusive education should be noted, which has led to the specific results derived from it being left out of the discussion. Thirdly, one of the main reasons small effect sizes were obtained in this study is the ceiling effect in the scores of attitudes and benefits. The small variability in the scores of attitudes and benefits makes it difficult to find differences, and, when they are found, such differences are likely to be large. Nevertheless, since families constitute an essential element in the setting of inclusive schools, future studies should delve into the factors related to the development of positive attitudes toward inclusion. Such contributions could help families become true catalysts of inclusion. But we must not forget that the educational inclusion of all students is a human right, so its defence is a responsibility of society as a whole.
